# Willingness of medical students to work on the COVID-19 frontline during the pandemic in China: A nationwide population-based cross-sectional study

**DOI:** 10.7189/jogh.14.05034

**Published:** 2024-12-20

**Authors:** Yuehui Jia, Yunfeng Han, Zhiping Xie, Xiaoting Chen, Wenting Li, Shuli Ma, Jun Wang, Jie Ge

**Affiliations:** 1 School of Public Health, Qiqihar Medical University, Qiqihar, China; 2 Sanitary Analysis Center, Scientific Research Office, Qiqihar Medical University, Qiqihar, China; 3 Office of educational administration affairs, Qiqihar Medical University, Qiqihar, China

## Abstract

**Background:**

The World Health Organization declared that coronavirus disease 2019 (COVID-19) constitutes an international public health emergency, which has strained health resources. In this study, we aimed to understand medical students' willingness to join the workforce fighting against the COVID-19 pandemic and identify factors associated with their decisions.

**Methods:**

We conducted a nationwide cross-sectional study using the Wen-Juan-Xing platform and a pre-designed questionnaire from 23 March to 19 April 2021. We conducted logistic regression analyses to identify the determinants associated with the willingness.

**Results:**

Among the 5022 medical students, the majority (n = 4289, 85.40%) expressed willingness to work on the COVID-19 frontline. Logistic regression indicated that medical students’ willingness to work on the COVID-19 frontline in China was associated with gender, region, reason for choosing medicine, having medical workers in the family, students whose family members, relatives or friends experienced COVID-19, and professional attitude. Females (odds ratio (OR) = 1.305; 95% confidence interval (CI) = 1.100–1.549; *P* = 0.0023), medical students from urban areas (OR = 1.295; 95% CI = 1.089–1.539; *P* = 0.0034), medical students whose choice of a medical career was their desire (OR = 1.579; 95% CI = 1.290–1.933; *P* < 0.0001), medical students whose parents or relatives are medical workers (OR = 1.266; 95% CI = 1.066–1.505; *P* = 0.0073), medical students whose family members, relatives, or friends have never been infected with COVID-19 (OR = 4.567; 95% CI = 3.002–6.947; *P* < 0.0001), and medical students with undisturbed of professional attitudes (OR = 4.280; 95% CI = 3.241–5.654; *P* < 0.0001) showed increased willingness to work on the COVID-19 frontline compared with their counterparts.

**Conclusions:**

Medical students demonstrated a strong willingness to contribute to COVID-19 work during the pandemic in China. The findings may provide valuable information for emergency management so that policymakers can maintain sufficient health resources and provide quality health care in similar health emergencies in the future.

The coronavirus disease 2019 (COVID-19) was an international public health emergency announced by the World Health Organization (WHO) on 31 January 2020 [1–4]. As of 4 August 2024, a total of 775 867 547 confirmed cases of COVID-19, including 7 057 145 deaths, have been reported to the WHO. As one of the many seriously affected countries, China has reported 99 373 219 confirmed cases of COVID-19, including 122 304 deaths [5,6]. The COVID-19 pandemic has led to significant disruptions to public life and health, while also straining health resources [7–11]. Hence, measures to provide quality health care and maintain sufficient health resources in major public health emergencies and scarcity of resources need to be studied.

Health professionals, especially physicians and nurses, were mainly involved in the treatment of confirmed cases of COVID-19. They also performed conventional medical examinations among people isolated because of close contact with confirmed COVID-19 cases, conducted nucleic acid tests for all residents in the pandemic areas, and led epidemiological investigations for contact tracing during the pandemic. Unfortunately, approximately 7% of all COVID-19 cases worldwide involve health professionals [12]. In China, more than 3000 medical workers have been infected with COVID-19, and more than 30 have died during the COVID-19 pandemic in March 2020 [13]. Therefore, the pandemic was not only a significant public health emergency, but also a major psychological crisis that may have altered the willingness of health professionals to work on the COVID-19 frontline [14–18]. A study conducted in Palestine reported that almost 25% of surveyed health care workers were unwilling to work during the COVID-19 pandemic [19]. Furthermore, up to 35.9% of 1051 health care workers in Nepal showed unwillingness to work on the COVID-19 frontline [20]. Similarly, another study found that 42% of health care workers in Australia were unwilling to work during the pandemic [21]. A meta-analysis revealed that the willingness of health care workers to work during an influenza pandemic ranged from 23.1% to 95.8% [22]. Therefore, during this global crisis, sufficient frontline health professionals had to have been maintained, and their willingness to work on the COVID-19 frontline during the pandemic needs to be investigated to mitigate the effect of COVID-19.

As both the current reserve and the future of the health system, medical students are expected to play an important role in the health care workforce [23]. Therefore, encouraging them to work on the frontline may help health systems handle the growing demands caused by a health crisis. This supportive strategy was implemented in Singapore and Hong Kong during the outbreaks of the severe acute respiratory syndrome in 2003 and in Saudi Arabia during the Middle East respiratory syndrome outbreak in 2014 [24–27]. The WHO Regional Office for Europe suggested that final-year students should be considered for employment, and volunteers should be called to increase the surge capacity in preparation for COVID-19 community transmission [28]. A study showed that 57.4% of 134 final-year medical students in Saudi Arabia were willing to join in the COVID-19 work during the pandemic, while another study in South Korea found that 66.3% of 315 medical students were willing to work on the COVID-19 frontline [29,30]. However, a nationwide assessment of the willingness of medical students to work on the COVID-19 frontline during the pandemic has not been conducted in China, which is among the many seriously affected countries by the COVID-19 pandemic. Therefore, under this international public health emergency, the willingness of medical students to work on the COVID-19 frontline needs to be explored to improve workforce deployment during similar health emergencies in the future in China.

Therefore, we aimed to explore the willingness of medical students in China to work on the COVID-19 frontline and identify the key factors associated with their decision. The findings of this study can provide valuable insights for policymakers to ensure sufficient health resources and alleviate the burden on the health care system during the COVID-19 pandemic. They can also help in preparing for future health crises by improving prevention and management strategies. From an educational perspective, understanding medical students’ willingness to engage in frontline work can inform curriculum development and enhance professional attitudes.

## METHODS

### Study design

We performed a nationwide population-based cross-sectional survey from 23 March to 19 April 2021 to investigate the willingness of medical students in China to work on the frontline during the COVID-19 pandemic and identify the determinants associated with their decision.

This study was approved by the ethical committee of Qiqihar Medical University (ref: (2021) 31) and adheres to the principles of the Helsinki Declaration. Participation in the research was voluntary, and data collection was conducted anonymously via an online platform. All participants provided informed consent online.

### Participants and investigation method

We conducted an anonymous online cross-sectional survey among medical students in mainland China using the Wen-Juan-Xing platform [31]. We used the snowball sampling method to recruit participants, whereby we created an electronic 2D code for the questionnaire through the online platform and distributed it to the counsellors by the investigators, categorised by universities, schools, majors, and grades. Subsequently, the counsellors forwarded the code to participants, who were also encouraged to recruit additional respondents by sharing the questionnaire.

Participation in this study was voluntary, and participants submitted the electronic questionnaire by scanning the code of the questionnaire with electronic equipment. Each IP address was configured to submit anonymously only once to prevent duplicate submissions. We marked all items in the questionnaire as mandatory to ensure data integrity.

Additionally, we considered responses invalid if the completion time was less than 180 seconds; this response time was automatically monitored by the Wen-Juan-Xing platform. The questionnaire did not include any identifying or sensitive content.

### Assessment tools and procedures

We administered a self-made questionnaire to participants (**Online Supplementary Document**). It consisted of 26 items, including socio-demographic information (13 items), professional attitudes (12 items), and the willingness of medical students to work on the frontline during the COVID-19 pandemic (one item). The sociodemographic information included gender, grade, major, the province where the family resides, the area of home located, only child, poor student, student leader, adjusting majors, reason for choosing medicine, medical workers in the family, internship experience, and family members or friends diagnosed with COVID-19. The professional attitudes of the medical students were assessed using a 12-item scale, with responses rated on a five-point scale: ‘very dissatisfied’ (1), ‘not satisfied’ (2), ‘basically satisfied’ (3), ‘satisfied’ (4), and ‘very satisfied’ (5). We calculated the total score by summing the scores of each item. The highest possible total score for professional attitudes was 60, with individual responses ranging from 12 to 60. The mean score for professional attitudes was determined by dividing the total score by 12. Additionally, a mean score of professional attitudes below 3 was classified as ‘disturbed’ professional attitudes, while a mean score of 3 or higher was classified as ‘undisturbed’. The willingness of medical students to work on the COVID-19 frontline was defined as binary variable (i.e. yes = 1, no = 0). The questionnaire was developed in accordance with the ‘Pneumonia Prevention and Control Plan for Novel Coronavirus Infection (Second Edition)’, the ‘Health Education Manual for Novel Coronavirus Pneumonia,’ and the ‘Guidelines for Public Protection of Novel Coronavirus Pneumonia’ [32–34]. Three experts in the field reviewed the questionnaire for content validity, after which it was revised and finally formalised. The reliability of the questionnaire was excellent, judging by a Cronbach’s alpha coefficient of 0.898.

### Statistical analysis

We performed the statistical analysis using SPSS, version 22.0 (SPSS Inc, Chicago, IL, USA). We expressed the categorical variables of the socio-demographic characteristics and the willingness as frequencies and percentages, and continuous variables of professional attitudes as mean and standard deviation (SD). We applied the Kolmogorov-Smirnov test to assess the normality of the variable distributions. We compared the difference of categorical variables between the groups using χ^2^ or Fisher exact probability tests, while normally distributed continuous variables were compared using *t*-tests. Lastly, we performed a logistic regression to identify the determinants associated with the willingness. The willingness of medical students to work on the COVID-19 frontline was treated as a dependent variable (yes = 1, no = 0). We expressed the risks of the determinants as odds ratios (OR) and the corresponding 95% confidence intervals (CI). We used the stepwise method to filter independent variables. Statistical significance was considered at a two-sided *P*-value <0.05.

## RESULTS

### Demographics and willingness to work on the COVID-19 frontline

**Figure 1:**
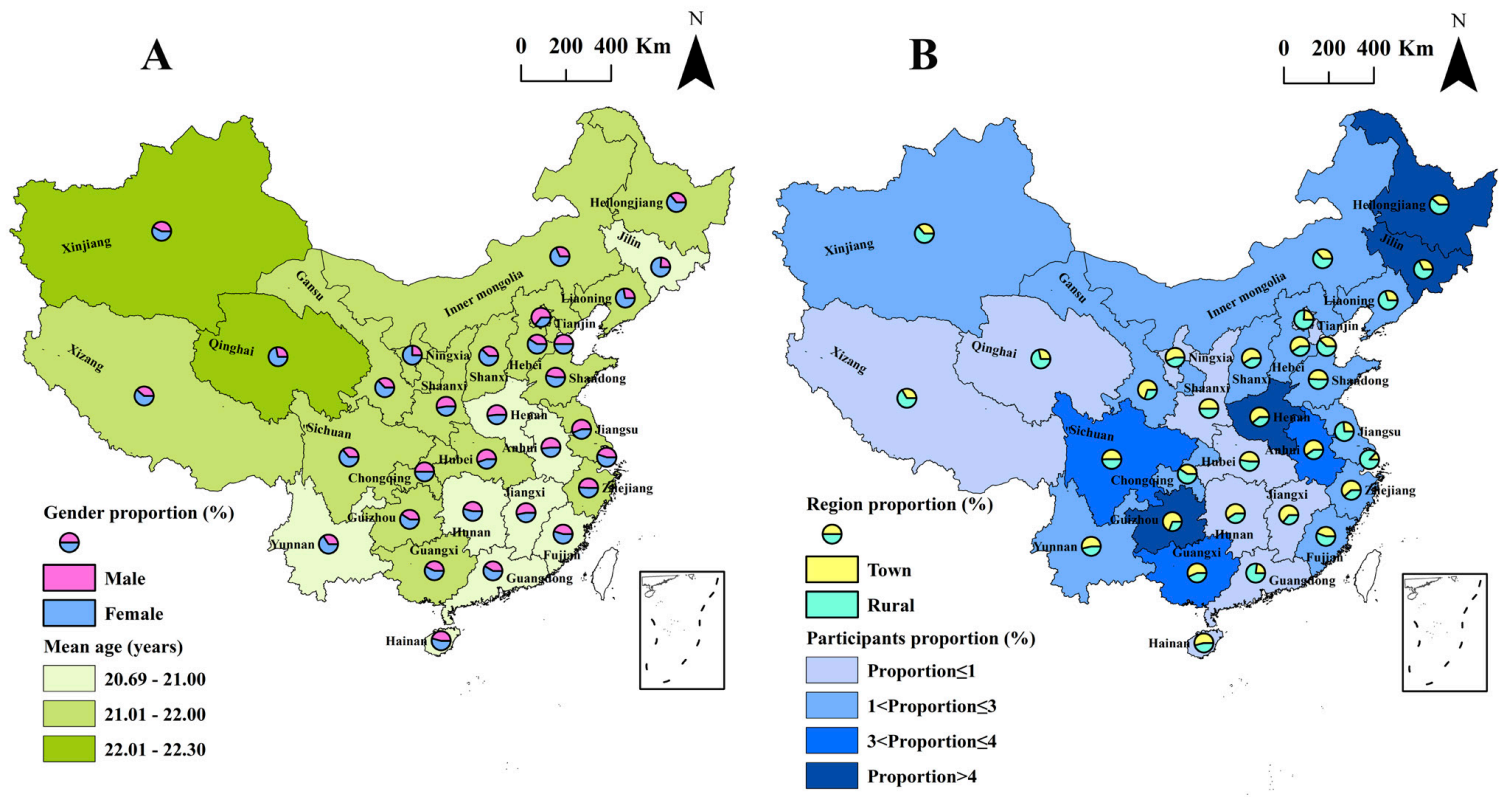
Spatial distribution of the participants by provinces. **Panel A.** Gender proportion and mean age of the subjects. **Panel B.** Region proportion and participants proportion of the subjects.

A total of 5069 medical students filled out the questionnaire. After removing incomplete responses, we included 5022 participants aged 15–27 years from 31 provinces in mainland China. The effective response rate was 99.07%. Of the 5022 participants, 1986 (39.55%) were male and 3036 (60.45%) female (**Figure 1**, Panel A). There were 2292 (45.64%) participants living in urban areas and 2730 (54.36%) living in rural areas (**Figure 1**, Panel B). Among the 5022 medical students, 4289 (85.40%) expressed willingness to work on the COVID-19 frontline during the pandemic in China, and 733 (14.60%) responded that they were unwilling.

A majority (86.82%) of females expressed willingness to work on the COVID-19 frontline, which was significantly higher than males (χ^2^ = 12.4281; *P* = 0.0004) (**Table 1**). Approximately 87.97% of first-grade, 86.35% of second-grade, 84.58% of third-grade, 82.84% of fourth-grade, and 83.60% of fifth-grade medical students showed a willingness to work on the COVID-19 frontline, and the difference was statistically significant (χ^2^ = 16.8752; *P* = 0.0020). On average, a significantly higher proportion of medical students living in urban areas (χ^2^ = 7.2410; *P* = 0.0071), being student leaders (χ^2^ = 4.3986; *P* = 0.0360), who had not adjusted their major (χ^2^ = 7.0174; *P* = 0.0081), pursuing a medical career out of their own desire (χ^2^ = 58.9970; *P* < 0.0001), having parents or relatives who are medical workers (χ^2^ = 6.1149; *P* = 0.0134), having no internship experience (χ^2^ = 3.9413; *P* = 0.0471), and not having family members, relatives, or friends infected with COVID-19 (χ^2^ = 87.4311; *P* < 0.0001) showed a willingness to fight on the frontline against COVID-19 compared to their counterparts.

**Table 1:** Demographic characteristics of medical students and their association with the willingness to work on the COVID-19 frontline

		Willingness to work			
**Variables by category**	**All, n (%)**	**Yes, n (%)**	**No, n (%)**	**χ^2^ value**	***P*-value**
Total	5022 (100%)	4289 (85.40)	733 (14.60)		
Gender				12.4281	0.0004
*Male*	1986 (39.55)	1653 (83.23)	333 (16.77)		
*Female*	3036 (60.45)	2636 (86.82)	400 (13.18)		
Grade				16.8752	0.0020
*First*	1446 (28.79)	1272 (87.97)	174 (12.03)		
*Second*	1121 (22.32)	968 (86.35)	153 (13.65)		
*Third*	629 (12.52)	532 (84.58)	97 (15.42)		
*Fourth*	1259 (25.07)	1043 (82.84)	216 (17.16)		
*Fifth*	567 (11.29)	474 (83.60)	93 (16.40)		
Major				0.1161	0.7333
*Clinical medicine*	2588 (51.53)	2206 (85.24)	382 (14.76)		
*Others*	2434 (48.47)	2083 (85.58)	351 (14.42)		
*Stomatology*	340 (6.77)	281 (82.65)	59 (17.35)		
*Anaesthesiology*	28 (0.56)	24 (85.71)	4 (14.29)		
*Preventive medicine*	402 (8.00)	359 (89.30)	43 (10.70)		
*Nursing medicine*	228 (4.54)	189 (82.89)	39 (17.11)		
*Medical imaging*	299 (5.95)	262 (87.63)	37 (12.37)		
*Medical examination*	133 (2.65)	117 (87.97)	16 (12.03)		
*Psychiatry*	486 (9.68)	409 (84.16)	77 (15.84)		
*Rehabilitation*	78 (1.55)	66 (84.62)	12 (15.38)		
*Other major*	440 (8.76)	376 (85.45)	64 (14.55)		
Region				7.2410	0.0071
*Urban areas*	2292 (45.64)	1991 (86.87)	301 (13.13)		
*Rural areas*	2730 (54.36)	2298 (84.18)	432 (15.82)		
Only child				2.8231	0.0929
*Yes*	2576 (51.29)	2179 (84.59)	397 (15.41)		
*No*	2446 (48.71)	2110 (86.26)	336 (13.74)		
Poor student identified by the school				2.0193	0.1553
*Yes*	1341 (26.70)	1161 (86.58)	180 (13.42)		
*No*	3681 (73.30)	3128 (84.98)	553 (15.02)		
Student leader				4.3986	0.0360
*Yes*	1451 (28.89)	1263 (87.04)	188 (12.96)		
*No*	3571 (71.11)	3026 (84.74)	545 (15.26)		
Major has been adjusted				7.0174	0.0081
*Yes*	544 (10.83)	444 (81.62)	100 (18.38)		
*No*	4478 (89.17)	3845 (85.86)	633 (14.14)		
The reason for choosing medicine				58.9970	<0.0001
*Own desire*	4128 (82.20)	3599 (87.19)	529 (12.81)		
*Suggested by others*	894 (17.80)	690 (77.18)	204 (22.82)		
Do your parents or relatives have medical workers?				6.1149	0.0134
*Yes*	2356 (46.91)	2043 (86.71)	313 (13.29)		
*No*	2666 (53.09)	2246 (84.25)	420 (15.75)		
Do you have internship experience?				3.9413	0.0417
*Yes*	2591 (51.59)	2188 (84.45)	403 (15.55)		
*No*	2431 (48.41)	2101 (86.43)	330 (13.57)		
Has anyone in your family, relatives or friends been diagnosed with COVID-19?				87.4311	<0.0001
*Yes*	109 (2.17)	59 (54.13)	50 (45.87)		
*No*	4913 (97.83)	4230 (86.10)	683 (13.90)		

### Professional attitudes and willingness to work on the COVID-19 frontline

**Figure 2:**
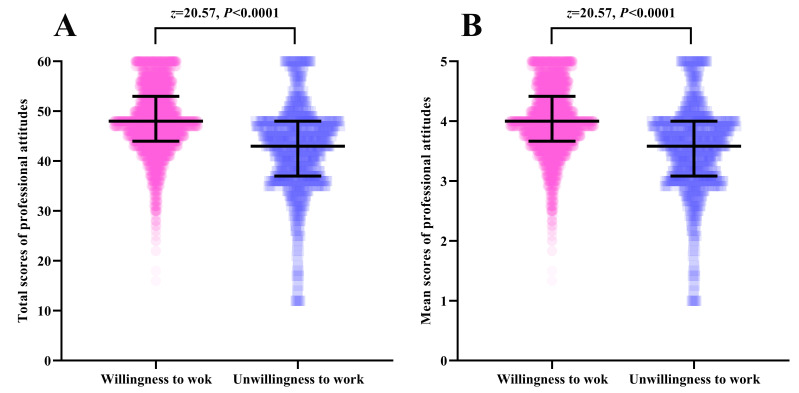
The distribution and z test of the scores of professional attitudes of medical students between willingness to work and unwillingness to work. **Panel A.** Total scores of professional attitudes. **Panel B.** Mean scores of professional attitudes.*Figures show means and standard deviations; the pies and squares represent the scores of professional attitudes of medical students in groups of willingness to work and unwillingness to work.

**Figure 3:**
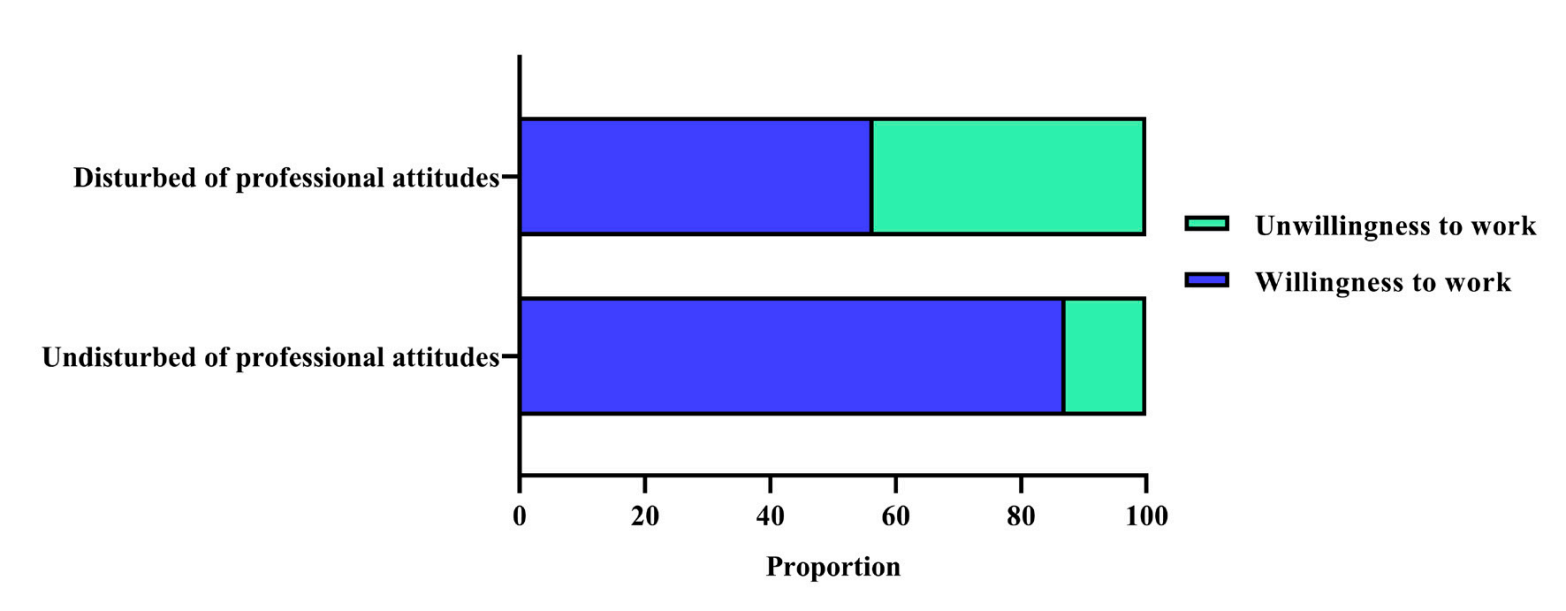
Proportions of willingness and unwillingness of medical students to work between undisturbed and disturbed professional attitudes.

The mean total score of professional attitudes of 5022 participants was 47.41 (SD = 7.43) (**Table 2**). The total scores of professional attitudes of 4289 participants who expressed willingness to work on the COVID-19 frontline were significantly higher than those who were unwilling (z = 20.57; *P* < 0.0001) (**Figure 2**, Panel A). The mean score of professional attitudes of 5022 participants was 3.95 (SD = 0.62). The mean scores of professional attitudes of 4289 participants who expressed willingness to work on the COVID-19 frontline were significantly higher than those who were unwilling (z = 20.57, *P* < 0.0001) (**Figure 2**, Panel B). Additionally, the participants were divided into two groups, namely ‘undisturbed of professional attitudes’ and ‘disturbed of professional attitudes,’ based on the mean scores of professional attitudes. There were 4751 (94.60%) participants with undisturbed professional attitudes and 271 (5.40%) participants with disturbed professional attitudes. Among these, a total of 87.06% of medical students with undisturbed professional attitudes showed a willingness to work on the COVID-19 frontline, which was significantly higher than the proportion of those with disturbed professional attitudes (χ^2^ = 192.55; *P* < 0.0001) (**Figure 3**).

**Table 2:** Professional attitudes of medical students and association with the willingness to work on the COVID-19 frontline

		Willingness to work			
**Variables by category**	**All (n = 5022)**	**Yes (n = 4289)**	**No (n = 733)**	Statistics*	***P-*value**
Continuous variable, x̄ (SD)				20.57	<0.0001
*Total scores of professional attitudes*	47.41 (7.43)	48.27 (6.84)	42.40 (8.67)		
*Mean scores of professional attitudes*	3.95 (0.62)	4.02 (0.57)	3.53 (0.72)		
Categorical variable, n (%)				192.55	<0.0001
*Undisturbed by professional attitudes*	4751 (94.60)	4136 (87.06)	615 (12.94)		
*Disturbed by professional attitudes*	271 (5.40)	153 (56.46)	118 (43.54)		

SD – standard deviation, x̄ – mean

*z-test was applied for continuous variables, while categorical variables were analysed using a χ^2^ test.

### Logistic regression of determinants for willingness to work on the COVID-19 frontline

**Figure 4:**
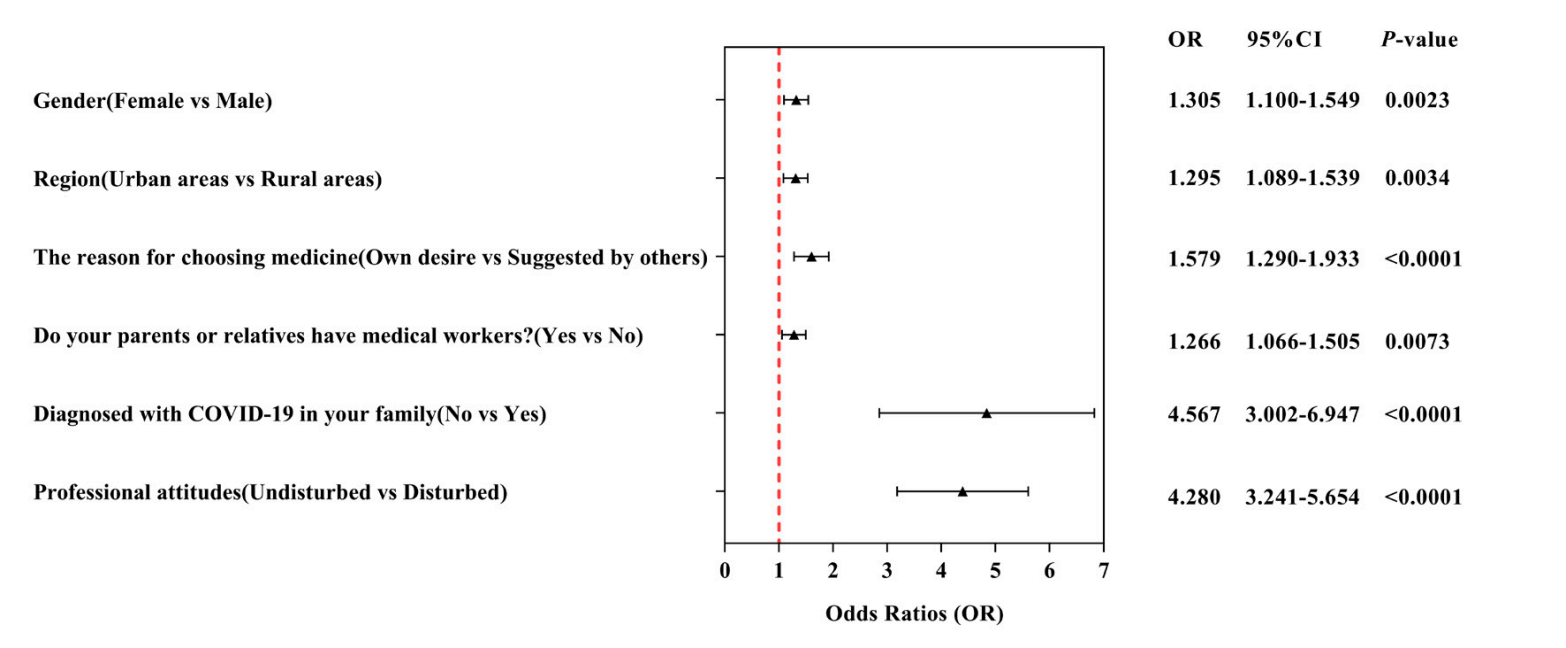
Logistic regression for analysis of the determinants significantly associated with the willingness to work on the COVID-19 frontline.

The willingness of medical students to work on the frontline during the COVID-19 pandemic was related to gender, region, reason for choosing medicine, having medical workers in the family, students whose family members, relatives, or friends experienced COVID-19, and professional attitude (**Figure 4****,** **Table 3**). Females had an increased willingness to work on the COVID-19 frontline compared to males (OR = 1.305; 95% CI = 1.100–1.549; *P* = 0.0023). Compared to their counterpart groups, medical students from urban areas (OR = 1.295; 95% CI = 1.089 − 1.539; *P* = 0.0034), those whose choice of a medical career was based on their desire (OR = 1.579; 95% CI = 1.290 − 1.933, *P* < 0.0001), those whose parents or relatives are medical workers (OR = 1.266; 95% CI = 1.066 − 1.505, *P* = 0.0073), those whose family members, relatives, or friends have never been infected with COVID-19 (OR = 4.567; 95% CI = 3.002 − 6.947, *P* < 0.0001), and those with undisturbed professional attitudes (OR = 4.280; 95% CI = 3.241 − 5.654, *P* < 0.0001) showed an increased willingness to work on the frontline.

**Table 3:** Logistic regression for analysis of the factors correlated with the willingness to work on the COVID-19 frontline*

Variables	β	SE	Wald χ^2^	*P-*value	OR (95% CI)
Gender					
*Male*	ref				
*Female*	0.2664	0.0873	9.3183	0.0023	1.305 (1.100–1.549)
Region					
*Rural areas*	ref				
*Urban areas*	0.2583	0.0881	8.5876	0.0034	1.295 (1.089–1.539)
*The reason for choosing medicine.*					
*Suggested by others*	ref				
*Own desire*	0.4571	0.1031	19.6456	<0.0001	1.579 (1.290–1.933)
Do your parents or relatives have medical workers?					
*No*	ref				
*Yes*	0.2361	0.0880	7.2048	0.0073	1.266 (1.066–1.505)
Has anyone in your family, relatives or friends been diagnosed with COVID-19?					
*Yes*	ref				
*No*	1.5189	0.2140	50.3678	<0.0001	4.567 (3.002–6.947)
Professional attitudes					
*Disturbed*	ref				
*Undisturbed*	1.4541	0.1420	104.9027	<0.0001	4.280 (3.241–5.654)

CI – confidence interval, OR – odds ratio, ref – reference, SE – standard error

*The willingness of medical students to work on the COVID-19 frontline treated as dependent variable (yes = 1, no = 0).

## DISCUSSION

This study is the first nationwide cross-sectional to investigate the willingness of medical students to work on the COVID-19 frontline during the pandemic in China and identify the determinants associated with their decision. During major public health emergencies, encouraging medical students to work on the frontline may be the quickest and most effective way to meet the growing demands of the health system and to maximise its ability to respond to the health emergency. Therefore, the willingness of medical students to join the COVID-19 work is an important research subject to improve workforce deployment during emergencies and provide valuable information to policymakers for maintaining sufficient health resources, relieving the burden on the health care system, and providing quality health care in similar health emergencies in the future.

Our findings revealed that 4289 out of 5022 (85.40%) of medical students expressed willingness to work on the COVID-19 frontline. It was higher than the 77 out of 134 (57.4%) reported in the study on the willingness of final-year medical students to work during the COVID-19 pandemic in Saudi Arabia [29], and that of 209 out of 315 (66.3%) reported in the study of medical and nursing students in South Korea [30]. Additionally, compared to other studies, our findings show that a higher percentage of medical students in China are willing to work on the COVID-19 frontline than primary care physicians in Taiwan (n/N = 428/625, 68.5%) [17], health care workers in Palestine (n/N = 268/357, 75.1%) [19], health care workers in Nepal (n/N = 674/1051, 64.1%) [20], health care workers in Australia (n/N = 337/580, 58.1%) [21], and hospital workers in South Korea (n/N = 265/441, 60.1%) [35]. However, our proportion is lower than that observed among nurses in Qatar (n/N = 332/377, 88.1%) [36], nurses in Wuhan Province, China (n/N = 1950/2014, 96.8%) [16], and nurses in Sichuan Province, China (n/N = 1185/1310, 90.5%) [12].

The higher willingness of medical students to work on the COVID-19 frontline in our study compared with previous studies can be explained by superior knowledge-attitude-practice regarding COVID-19, psychological status during the pandemic among college students in China [10,37], and firm professional attitudes of medical students found in the present study [38]. The National Health Commission released and disseminated knowledge and prevention and control measures regarding the COVID-19 pandemic to the public through media and online platforms and established a real-time reporting system during the COVID-19 pandemic. All these measures implemented by the Chinese government may have effectively bolstered public self-protective awareness and confidence in overcoming the COVID-19 pandemic, thus increasing the willingness of medical students in China to work on the COVID-19 frontline.

The results of the logistic regression indicated that females showed a greater willingness to work on the COVID-19 frontline compared to males (OR = 1.305). Other studies, specifically those involving nurses from Southwestern China, primary care physicians from Taiwan, and health care workers from Palestine, have shown no significant gender differences in the willingness of health workers to participate in COVID-19 work [12,16,17,19]. However, a study regarding COVID-19-related knowledge, attitude, and practice among college students in China showed that female students scored significantly higher in COVID-19-related knowledge than males [37]. Furthermore, a pre-post-like study among medical students in China regarding the impact of the COVID-19 pandemic on professional attitudes confirmed that the proportion of female medical students with disturbed professional attitudes was significantly lower than that of males [38]. Therefore, these studies support our findings that females show a greater willingness to work on the COVID-19 frontline compared to males. The reasons for this may include higher scores in COVID-19-related knowledge and a stronger professional attitude among female students, which could enhance their attitude and practice in fighting the COVID-19 pandemic, thereby increasing their willingness to work on the frontline. Therefore, our results suggest that female medical students may be more willing to join in the frontline work in similar public health emergencies than males, while male medical students should be further encouraged.

Medical students from urban areas had an increased willingness to work on the COVID-19 frontline compared to those from rural areas (OR = 1.295). Previous studies have indicated that knowledge scores regarding COVID-19 were higher among students from urban areas than those from rural areas, and the proportion of medical students from urban areas with disturbed professional attitudes was significantly lower than those from rural areas [37,38]. A heightened level of knowledge and a stable professional attitude regarding COVID-19 may enhance the willingness of medical students to work on the COVID-19 frontline. Therefore, it is essential to improve the knowledge level and professional attitude of medical students in preparation for similar public health emergencies.

Medical students whose family members, relatives, or friends had never been infected with COVID-19 expressed a greater willingness to work on the COVID-19 frontline compared to those whose family members, relatives, or friends had been infected (OR = 4.567). One systematic review highlighted that the general public with relatives infected by COVID-19 was at an increased risk of developing psychiatric symptoms, including anxiety and depression [39]. Furthermore, a cross-sectional study regarding the psychological status of Chinese college students during the COVID-19 pandemic found that students with anxiety symptoms had significantly lower scores in knowledge, attitude, and practice regarding COVID-19 than those without anxiety symptoms [10]. These findings indicate that medical students who had family members, relatives, or friends infected with COVID-19 may be more susceptible to psychological disorders such as anxiety and depression, which could lead to lower scores in knowledge, attitude, and practice regarding COVID-19, ultimately affecting their willingness to work on the frontline. Therefore, educational institutions must prioritise the mental health of medical students during similar public health emergencies, especially for those with family members, relatives, or friends infected by COVID-19, to enhance their willingness to work on the frontline.

The increased willingness of medical students to work on the COVID-19 frontline was also associated with their desire to choose medicine (OR = 1.579), having parents or relatives who are medical workers (OR = 1.266), and their strong professional attitudes (OR = 4.280). These findings suggest that these medical students may possess a substantial understanding of their field, a deep passion for their major, strong professional attitudes, and a commitment to professionalism. The attitudes of medical students toward their profession not only directly affect their development of far-reaching professional ideals and the cultivation of noble professional ethics during their studies, but also affect their ability to maintain professional commitment and uphold good professional ethics in the future, once they become fully qualified medical workers. Therefore, the professional attitudes of medical students may be a significant factor associated with their willingness to work on the COVID-19 frontline during the pandemic. Consequently, it is essential to enhance the professional attitudes of medical students, especially those whose medical careers are primarily influenced by external recommendations and those who lack medical professionals in their families, during similar public health emergencies.

### Strengths and limitations

This was the first nationwide cross-sectional study in China to investigate the willingness of medical students to work on the COVID-19 frontline, identify the determinants associated with their decision, and explore the association between professional attitudes and this willingness. Therefore, it can inform workforce deployment during emergencies and provide valuable information to policymakers to maintain sufficient health resources in future similar health emergencies. The major limitation of this study was the use of non-probability sampling, which may have induced selection bias. Therefore, the willingness to work on the COVID-19 frontline among Chinese medical students who were not included in the study could not be evaluated.

## CONCLUSIONS

The majority of medical students in our study were willing to join in the COVID-19 work during the pandemic in China. Medical students who were females, from urban areas, whose medical career was their desire, whose parents or relatives are medical workers, whose family members, relatives, or friends have never been infected with COVID-19, and those with undisturbed of professional attitudes showed increased willingness to work on the COVID-19 frontline compared to their counterparts. These findings may provide valuable information to emergency management for policymakers to maintain sufficient health resources and to provide quality health care in future similar health emergencies.

## Additional material


Online Supplementary Document
