# Supplementary material for: Willingness of medical students to work on the COVID-19 frontline during the pandemic in China: A nationwide population-based cross-sectional study
Source: J Glob Health. 2024 Dec 20;14:05034. doi: 10.7189/jogh.14.05034 (PMC11658715; doi:10.7189/jogh.14.05034)
Supplement: Online Supplementary Document [file jogh-14-05034-s001.pdf]

Willingness of medical students to work on the COVID-19 frontline during the pandemic in China: A nationwide population-based cross-sectional study

Dear students:

This survey answers questions regarding the willingness of medical students to participate in the frontline work during the COVID-19 pandemic in China. Please answer the following questions herein according to your own experiences or actual situations. This survey is completely anonymous. Your real thoughts will help us immensely. Thank you for your kind help.

Basic information

1. Gender:

   A. Male

   B. Female

2. Grade:

   A. First

   B. Second

   C. Third

   D. Fourth

   E. Fifth

3. Major:

   A. Clinical medicine

   B. Stomatology

   C. Anesthesiology

D. Preventive medicine

E. Nursing medicine

F. Medical imaging

G. Medical Examination

H. Psychiatry

I. Rehabilitation

J. Other major

4. What province does your family resides?

_________________ province.

5. Where is your home located?

A. Urban area

B. Rural area

6. Are you an only child in your family?

A. Yes

B. No

7. Are you a poor student identified by the school?

A. Yes

B. No

8. Are you a student leader?

A. Yes

B. No

9. Has your major been adjusted?

A. Yes

B. No

10. The reason for choosing medicine.

A. Own desire

B. Suggested by others

11. Do your parents or relatives have medical workers?

A. Yes

B. No

12. Do you have internship experience?

A. Yes

B. No

13. Has anyone in your family, relatives or friends been diagnosed with COVID-19?

A. Yes

B. No

Professional attitudes

14. Do you think the doctor-patient relationship will improve?

A. Strongly disagree

B. Disagree

C. Basically agree

D. Agree

E. Strongly agree

15. Would you like to continue your medical career?

A. Strongly disagree

B. Disagree

C. Basically agree

D. Agree

E. Strongly agree

16. If you could choose again, would you still choose to study medicine?

A. Strongly disagree

B. Disagree

C. Basically agree

D. Agree

E. Strongly agree

17. Are you proud of being a medical student?

A. Strongly disagree

B. Disagree

C. Basically agree

D. Agree

E. Strongly agree

18. What is your attitude towards “I am very optimistic about the employment prospects of my major?”

A. Strongly disagree

B. Disagree

C. Basically agree

D. Agree

E. Strongly agree

19. Do you think the income of medical staff is proportional to the effort required?

A. Strongly disagree

B. Disagree

C. Basically agree

D. Agree

E. Strongly agree

20. Do you think it is necessary to continue treatment for the person who injured or assaulted the doctor?

A. Strongly disagree

B. Disagree

C. Basically agree

D. Agree

E. Strongly agree

21. Do you think the work of medical staff is respected and recognized by society?

A. Strongly disagree

B. Disagree

C. Basically agree

D. Agree

E. Strongly agree

22. As a medical student, you think your future career is “healing the wounded, lofty and sacred.”

A. Strongly disagree

B. Disagree

C. Basically agree

D. Agree

E. Strongly agree

23. I firmly believe that I can become an excellent medical worker in the future.

A. Strongly disagree

B. Disagree

C. Basically agree

D. Agree

E. Strongly agree

24. I was mentally prepared for a career in medicine.

A. Strongly disagree

B. Disagree

C. Basically agree

D. Agree

E. Strongly agree

25. My ideal career would be in medicine.

A. Strongly disagree

B. Disagree

C. Basically agree

D. Agree

E. Strongly agree

Willingness

26. Are you willing to work in the frontline during the pandemic?

A. Yes

B. No

We promise that all information will not be shared elsewhere and will only be used for this scientific research. Thank you again for your participation. Finally, I wish you good health, academic success and a smooth life.
